# Cardiovascular Magnetic Resonance Imaging of Myocardial Interstitial Expansion in Hypertrophic Cardiomyopathy

**DOI:** 10.1007/s12410-014-9267-z

**Published:** 2014-03-29

**Authors:** Timothy C. Wong

**Affiliations:** Division of Cardiology, Department of Medicine, University of Pittsburgh School of Medicine, UPMC Hypertrophic Cardiomyopathy Center, UPMC Cardiovascular Magnetic Resonance Center, Heart and Vascular Institute, University of Pittsburgh Medical Center, A-344 Scaife Hall, 200 Lothrop St., Pittsburgh, PA 15213 USA

**Keywords:** Hypertrophic cardiomyopathy, Myocardial fibrosis, Cardiovascular magnetic resonance, T1 mapping, Extracellular volume fraction, Gadolinium contrast

## Abstract

Hypertrophic cardiomyopathy (HCM) is a cardiovascular genetic disease with a varied clinical presentation and phenotype. Although mutations are typically found in genes coding for sarcomeric proteins, phenotypic derangements extend beyond the myocyte to include the extracellular compartment. Myocardial fibrosis is commonly detected by histology, and is associated with clinical vulnerability to adverse outcomes. Over the past decade, the noninvasive visualization of myocardial fibrosis by cardiovascular magnetic resonance (CMR) techniques has garnered much interest given the potential applications toward improving our understanding of pathophysiologic mechanisms of disease, as well as diagnosis and prognosis. Late gadolinium enhancement (LGE) imaging techniques are able to detect focal (typically replacement) fibrosis. Newer CMR techniques that measure absolute T1 relaxation time allow the quantification of the entire range of focal to diffuse (interstitial) fibrosis and may overcome potential limitations of LGE. This review will discuss the methodology and current status of these novel techniques, with a focus on extracellular volume fraction (ECV). Recent findings describing ECV measurement in HCM will be summarized.

## Introduction

Hypertrophic cardiomyopathy (HCM) is the most common genetic cardiovascular disorder, with a prevalence of 0.2 % reported across several regions of the world [1–3], yet current management strategies lack therapy directly targeting the underlying disease process(es). The genetic basis is currently attributed to over 1400 unique mutations in at least 11 genes coding for proteins comprising the sarcomere [4]. Clinically, the diagnosis is usually determined by the identification of left ventricular hypertrophy considered unlikely to be caused by another cardiac or systemic condition [1]. Alternatively, genotyping may determine the presence of a mutation deemed pathogenic. The phenotypic manifestations and clinical course are heterogeneous, likely reflecting not only the diversity of mutations, but additional interactions with modifier genes and environmental influences. The treatment of symptoms such as angina or dyspnea on exertion hinges on the determination of left ventricular outflow tract obstruction due to hypertrophied myocardium and mitral valve systolic anterior motion. Such obstruction can be palliated with anti-inotropic medication and with invasive intervention (surgical myectomy or alcohol ablation of the hypertrophied septum) if symptoms remain refractory to maximal medical therapy. Heart transplantation is also available for those who develop severe heart failure. While HCM is also associated with malignant arrhythmias and sudden death, those identified as higher risk can be treated with implantable defibrillator therapy [1]. However, risk stratification remains an imprecise science, with infrequent appropriate defibrillator discharge rates [5] accompanying the reduction in population risk. There is a clinical need to move beyond palliation and risk reduction and target fundamental disease pathways.

Myocardial fibrosis is increasingly recognized as an intrinsic phenotype of HCM. Although mutations are typically located in genes coding for sarcomeric proteins, phenotypic derangements extend beyond the myocyte to include the extracellular compartment. Indeed, the 2010 National Heart Lung and Blood Institute HCM Working Group [6] noted the lack of data regarding “extra-cardiomyocyte manifestations of HCM” and called for research that would identify and characterize mechanisms mediating fibrosis and remodeling. The advent of cardiovascular imaging techniques capable of visualizing myocardial fibrosis permits routine, noninvasive characterization of the interstitial space previously limited to biopsy and postmortem analysis. Such information would not only improve the understanding of fundamental disease pathogenesis, but potentially illuminate therapeutic targets. This article will briefly review the histopathologic characterization of fibrosis in HCM, CMR late gadolinium enhancement detection of macroscopic fibrosis and its limitations, and then focus on emerging CMR T1 quantification techniques. which can detect the entire spectrum of fibrosis from focal to diffuse. Finally, we will summarize recent data characterizing ECV among those with HCM.

## Myocardial Fibrosis in Hypertrophic Cardiomyopathy

Myocardial fibrosis is commonly observed in hypertrophic cardiomyopathy, and has been associated with a range of clinical outcomes. There are various forms of such fibrosis, often categorized as replacement and interstitial. Replacement fibrosis refers to the accumulation of collagen where myocyte damage and/or necrosis has occurred [7, 8], and may be focal or diffuse. Interstitial fibrosis occurs more diffusely throughout the extracellular space of the myocardium, although it can form along perivascular bundles as well [7, 8]. The mechanisms driving interstitial fibrosis in HCM are incompletely understood, but are thought to be mediated in part by transforming growth factor β1, which triggers the production of extracellular matrix proteins [9]. Although precise definitions of replacement or interstitial fibrosis may vary somewhat according to the method of detection (eg, histology vs imaging), derangement of the extracellular compartment has been increasingly recognized as a predictor of outcomes including sudden death, ventricular arrhythmias, and heart failure. Basso et al [10] and Shirani et al [11] both demonstrated a high prevalence of fibrosis detected at autopsy among young patients who suffered sudden death. In the latter study, qualitative assessment of interstitial collagen was performed using picrosirius red (a collagen specific stain) as well as quantitative collagen volume fraction determination by semi-automated image analysis techniques. Of note, areas of disorganized myocytes (termed myocardial disarray) were also observed and quantified. Varnava et al also described similar findings among those with end-stage heart failure [12].

Given the invasive nature of histology for fibrosis detection and quantification, the ability of noninvasive imaging methods to visualize extracellular compartment expansion has garnered much interest. Various non-CMR modalities for such detection have been previously reviewed [13].

## Noninvasive Detection of Focal Fibrosis in HCM

Late gadolinium enhancement (LGE) imaging techniques utilize gadolinium contrast agents, which distribute preferentially to myocardial regions containing extracellular compartment expansion following a bolus. Although initially used for detection of myocardial infarction (dense regions of replacement fibrosis confirmed by robust histologic validation), LGE techniques were also found to identify atypical signal patterns thought to represent scar of nonischemic etiology. Among those with HCM, Choudhury et al [14] described patchy patterns of multiple foci of abnormal LGE signal in 17 of 21 patients. The histologic validation of atypical LGE has been less robust compared with that of myocardial infarction, but small studies have provided some corroboration. Moon et al [15] demonstrated high correlation between areas of LGE and scar by histology in a single heart obtained following heart transplantation. A larger series by Moravsky et al [16] demonstrated good correlation between quantitative assessment of histologic fibrosis and LGE in myocardial samples obtained at the time of septal myectomy surgery. Reassuringly, CMR identification of LGE has been linked with important patient outcomes of sudden death, arrhythmia, and heart failure in multiple centers [17–20].

There are, however, limitations of the LGE technique that are important to consider, especially in the HCM arena [21]. Given the high prevalence of LGE among those with HCM [22], there is a clinical need to move beyond binary reporting of its presence or absence and to quantify precisely its extent. There is no universally accepted strategy for such quantification, in part due to the variety of algorithms proposed—each of which have strengths and weaknesses [23] (eg, manual tracing vs full-width at half-maximum vs various standard deviations beyond a normal region of interest). In addition, the heterogeneous nature of fibrosis accumulation in HCM ranging from focal to diffuse may preclude the availability of a completely “normal” region of myocardium to act as a reference. Other factors leading to variability in quantification may be considered by category, including: *image acquisition* parameters (spatial resolution, slice thickness, segmented vs single shot imaging, phase sensitive vs magnitude reconstruction, and the time elapsed between bolus and LGE imaging), *patient* parameters (heart rate, gadolinium concentration dependence on weight based dosing, and glomerular filtration rate), and also *contrast agent* characteristics (protein binding, relaxivity, and dosing strategy—especially important, given a nonlinear relationship between signal intensity and gadolinium concentration).

Recent advances in CMR imaging which allow for extracellular volume fraction (ECV) quantification may offer solutions to many of these limitations. ECV takes advantage of absolute measurement of T1 relaxation time, compared with relative differences in T1 weighted signal intensity for LGE imaging, and can detect the entire range of fibrosis from focal to diffuse.

## Noninvasive Detection of Diffuse Fibrosis in HCM

The development of CMR techniques which perform rapid, serial experiments by which the absolute T1 time of tissue, and subsequently the extracellular volume fraction, may be derived has generated considerable interest. A brief discussion of terminology in this rapidly evolving field is relevant to this review. *Native T1* refers to myocardial T1 measurement without the use of gadolinium contrast agents, and reflects myocardial characteristics affecting both the cellular and extracellular compartment [24••]. For example, shortened *native T1* is observed due to intracellular lipid accumulation in Anderson-Fabry disease [25, 26], a phenocopy of HCM. *Postcontrast T1* refers to T1 measurement at a time point after gadolinium administration (typically bolus), which again may reflect myocardial properties. However, *postcontrast T1* may be confounded by variations in the precise time of measurement, body weight, and corresponding contrast dose, rate of gadolinium clearance (dependent on renal function), and hematocrit [27••]. Finally, the *extracellular volume fraction* (ECV) technique directly measures the proportion of myocardium occupied by extracellular space—typically a marker of fibrosis in the absence of edema or amyloid deposition. ECV exploits the extracellular nature of gadolinium and measures myocardial uptake of contrast relative to plasma since gadolinium and plasma equilibrate during slow renal washout. The proportion of uptake can be computed by the change in relaxivity (inverse of T1) for myocardium and plasma since the change in relaxivity linearly relates to gadolinium concentration. Plasma change in relaxivity is derived from blood change in relaxivity by multiplying the latter by (1-hematocrit). Because the HCM phenotype is most noted for extracellular fibrosis (as opposed to intracellular derangement), and because of inherent variability in postcontrast T1 measurement, this article will focus on ECV measurement as a marker of extracellular matrix expansion and fibrosis.

**Fig. 1:**
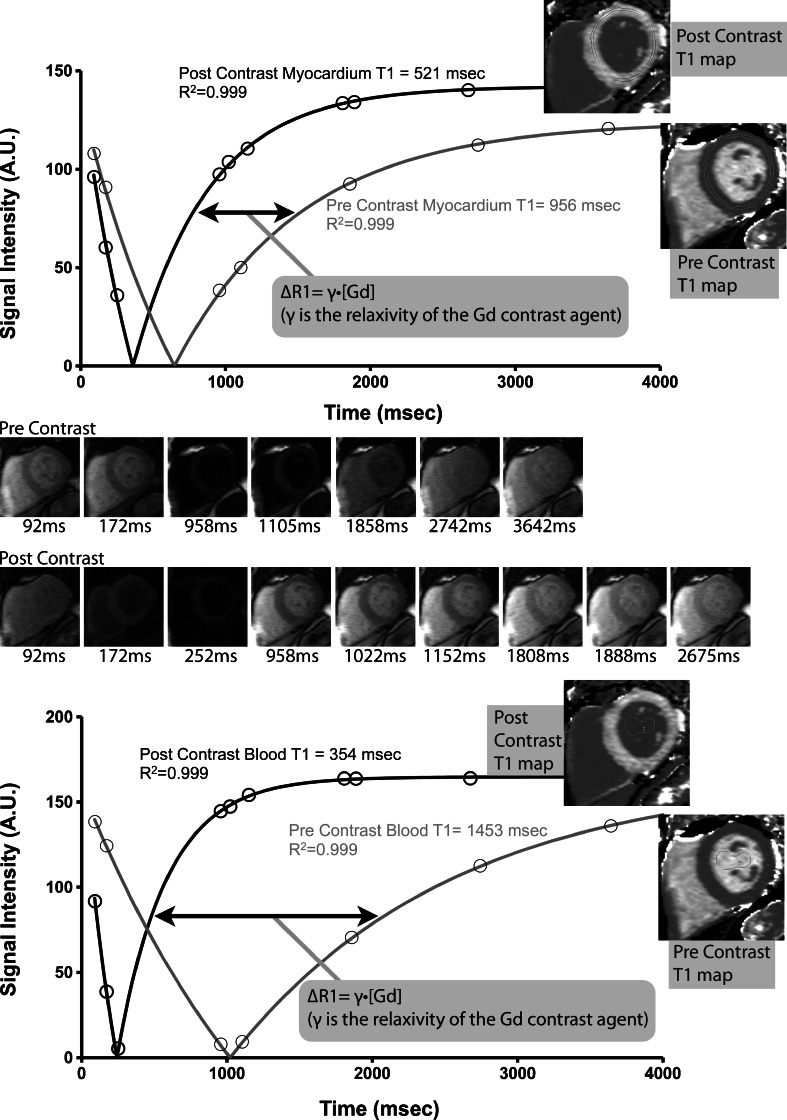
(Adapted from Wong et al [32], with permission from Lippincott Williams & Wilkins - Wolters Kluwer Health). Measurement of pre and postcontrast T1 in an individual with normal ECV. Short axis imaging at varying time points (precontrast images top row, postcontrast images bottom row) along with the corresponding fitting curves used to derive absolute T1 measurement. Calculations are performed at the pixel level to generate pre and postcontrast T1 maps

There are various methods for absolute T1 measurement, which have coalesced along 3 main techniques which involve either inversion recovery or saturation recovery experiments: Modified Look-Locker Inversion (MOLLI) recovery [28], Shortened MOLLI (ShMOLLI) [29], and Saturation Recovery Single-shot Acquisition (SASHA) [30]. A detailed description and comparison of these techniques is beyond the scope of this article, although a cogent discussion was recently published [31]. Figure 1 demonstrates a sample set of imaging experiments used to obtain myocardial and blood T1 measurement in a normal individual. Once a method for T1 measurement is set, motion-correction of serial imaging experiments and then mapping may be performed on a pixel-wise basis so that a T1 map is generated such that each pixel reflects the absolute T1 value of that region [27••].

**Fig. 2:**
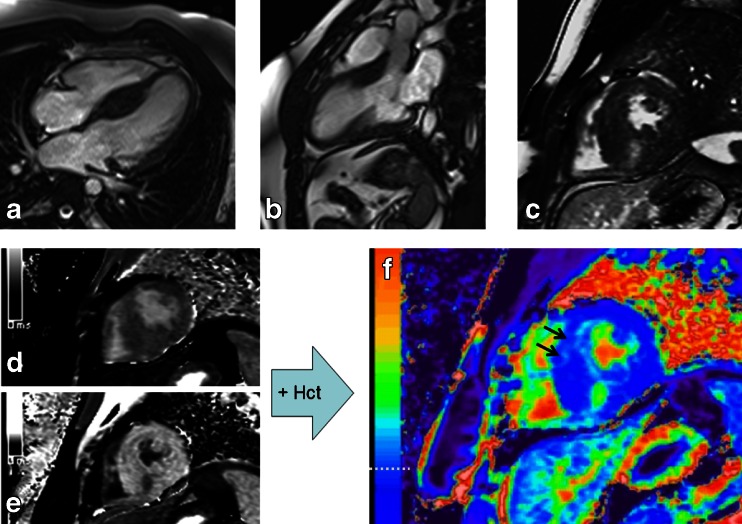
CMR images of a patient with hypertrophic cardiomyopathy. **a** Four-chamber steady state free precession (SSFP) image demonstrating asymmetric septal hypertrophy. **b** Three-chamber SSFP image. **c** Midventricular short axis LGE image ~15 minutes postgadolinium contrast showing focal LGE in the vicinity of the right ventricular insertion point. **d** Precontrast T1 map. **e** postcontrast T1map ~ 20 minutes postgadolinium contrast. **f** ECV map calculated using the T1 data and hematocrit. The horizontal dashed white line notes the color range of the upper limit of normal of ECV (~29.5 % at our center). The black arrows point to the anterior septal region where more diffuse fibrosis is identified, which was not as readily apparent on the LGE image (panel (**c**). Note: panels **c**, **d**, **e**, **f** are the same short axis slice

Using T1 values of myocardium and blood, obtained pre- and postcontrast, ECV measurement may be performed to obtain an estimate of the % fraction of extracellular space in the tissue of interest. In brief, ECV = λ * (1–hematocrit) where λ is the partition coefficient for gadolinium in the myocardium and blood pool, which is equal to the ratio of the corresponding change in relaxivity (∆R) before and after contrast administration (λ = ∆R1myocardium / ∆R1blood, ∆R1 = 1/T1postGd–1/T1preGd). A more detailed description of the calculation steps has been previously described [32]. Furthermore, T1 mapping data may be used to compute ECV maps. Figure 2 demonstrates a representative HCM imaging case from our center where T1 maps are acquired pre and postcontrast, and used (along with the hematocrit) to produce an ECV map. Motion correction algorithms maximize image coregistration required for pixelwise parametric maps of T1 and ECV [33].

The histologic validation of ECV and diffuse myocardial fibrosis in HCM has been described by several centers. Flett et al [34] reported excellent correlation (R^2^ = 0.86) between ECV and collagen volume fraction in a cohort of patients with either aortic stenosis or hypertrophic cardiomyopathy. Fontana et al [35] and White et al [36] both demonstrated good correlations with quantitative histology using variations on the ECV technique in a wider spectrum of patients with aortic stenosis, hypertrophic cardiomyopathy, amyloid, chronic myocardial infarction. Miller et al also described robust agreement in an elegant, comprehensive study of whole heart validation of ECV against histology [37] in hearts explanted from humans at the time of heart transplantation. Validation of ECV against longitudinal outcomes prediction among those with HCM has not been published to date, although our group has demonstrated incremental prognostic value of ECV for clinical outcomes in a large cohort, which did not include HCM [32, 38].

While such strong validation data are certainly encouraging, several words of caution regarding interpretation of ECV data should be mentioned. It is important to note that ECV reflects measurement of the entire extracellular space and does not inform regarding potential variables of interest such as the state of collagen cross-linking or extent of signal due to myocardial disarray. Furthermore, heterogeneity remains regarding standardization of the measurement technique (platform, choice of gadolinium chelate, etc). Fortunately, a T1 mapping working group has convened to produce a recent consensus document [24••] summarizing agreement and challenges in the field, with plans for further updates as the field continues to mature.

## Current Studies of ECV in HCM

Already, several centers have begun exploring the role of ECV as a novel imaging biomarker of diffuse myocardial fibrosis among those with HCM. From a diagnostic standpoint, Ugander et al [39], Sado et al [40], and Kellman et al [41], all demonstrated the ability of abnormally elevated ECV measurement to discriminate between myocardial regions (and individuals) of health vs disease. Our group has observed a preliminary association between ECV and BNP [42], of interest given the recent observation that BNP may be a relevant HCM disease severity marker [43]. Also, Ho et al [8] report the observation that ECV is abnormally elevated in sarcomeric HCM mutation carriers without left ventricular hypertrophy, a finding with implications both for diagnosis as well as assessment of disease response to anti-fibrotic therapies. Finally, we await the results of the large observational study of HCM recently organized by Kramer and Neubauer to characterize prognostic markers in HCM, including T1 mapping markers of diffuse fibrosis (ClinicalTrials.gov identifier NCT01915615).

## Conclusions

Myocardial fibrosis, both focal replacement as well as diffuse interstitial types, is prevalent in HCM and related to adverse clinical outcomes. The noninvasive capability of contrast enhanced CMR imaging techniques allows for identification and serial monitoring of this important disease phenotype. The advent of absolute T1 measurement techniques permit the measurement of extracellular volume fraction, which can quantify the entire range of fibrosis from diffuse to focal, and may overcome some limitations of current late gadolinium enhancement based techniques. The application of ECV to the study of HCM is nascent, and much further work remains to be done to determine the role of ECV in diagnosis, prognosis, and assessment of disease response to targeted therapy.
